# Seed coat thinning during horsegram (*Macrotyloma uniflorum*) domestication documented through synchrotron tomography of archaeological seeds

**DOI:** 10.1038/s41598-017-05244-w

**Published:** 2017-07-14

**Authors:** Charlene Murphy, Dorian Q. Fuller

**Affiliations:** University College London, Institute of Archaeology, London, WCH1 0PY UK

## Abstract

Reduction of seed dormancy mechanisms, allowing for rapid germination after planting, is a recurrent trait in domesticated plants, and can often be linked to changes in seed coat structure, in particular thinning. We report evidence for seed coat thinning between 2,000 BC and 1,200 BC, in southern Indian archaeological horsegram (*Macrotyloma uniflorum*), which it has been possible to document with high precision and non-destructively, through high resolution x-ray computed tomography using a synchrotron. We find that this trait underwent stepped change, from thick to semi-thin to thin seed coats, and that the rate of change was gradual. This is the first time that the rate of evolution of seed coat thinning in a legume crop has been directly documented from archaeological remains, and it contradicts previous predictions that legume domestication occurred through selection of pre-adapted low dormancy phenotypes from the wild.

## Introduction

Crop domestication is a co-evolutionary process which adapted plants to cultivation and facilitated the growth of larger sedentary human populations^1, 2^. The documentation of domestication in the archaeological record is often hampered by limited preservation of diagnostic morphology in archaeological plant specimens, with seed dispersal traits preserved in some cereals and grain size information in a wider range of taxa^3, 4^. Reduction of seed dormancy mechanisms, allowing for rapid germination after planting, is a recurrent trait in domesticated plants, and can often be linked to changes in seed coat structure, in particular thinning^5^, but this has been documented previously in sub-fossil material of only a few taxa, such as New World *Chenopodium* spp.^4, 6, 7^. In pulses (domesticated Fabaceae seed crops) reduction of seed dormancy is regarded as central to domestication as wild legumes have conspicuous dormancy related to thick, hard seed coats^8–11^. In some legumes, such as pea (*Pisum sativum*) the reduction in dormancy is linked to a conspicuous reduction in seed coat sculpturing^9^, but this is by no means universal across pulses. It has been inferred that only rare, wild plants that had reduced dormancy would have been suitable for early cultivators to select from the wild for cultivation^8, 12, 13^. However, archaeobotanical evidence documenting the presence of thin-seed coated, low dormancy phenotypes prior to or at the beginnings of cultivation has never been reported, nor have archaeobotanists previously documented evidence for the evolution, whether rapid or gradual, of pulse testa morphology. While this has been discussed largely in relation to Southwest Asian domesticates such a lentil (*Lens culinaris*) and pea^8–10, 12, 13^, it should apply equally across all pulses. Using a novel methodology to non-destructively document seed coat thickness around entire archaeological pulse seeds, we report data from the South Asia pulse crop horsegram (*Macrotyloma uniflorum*).

**Figure 1:**
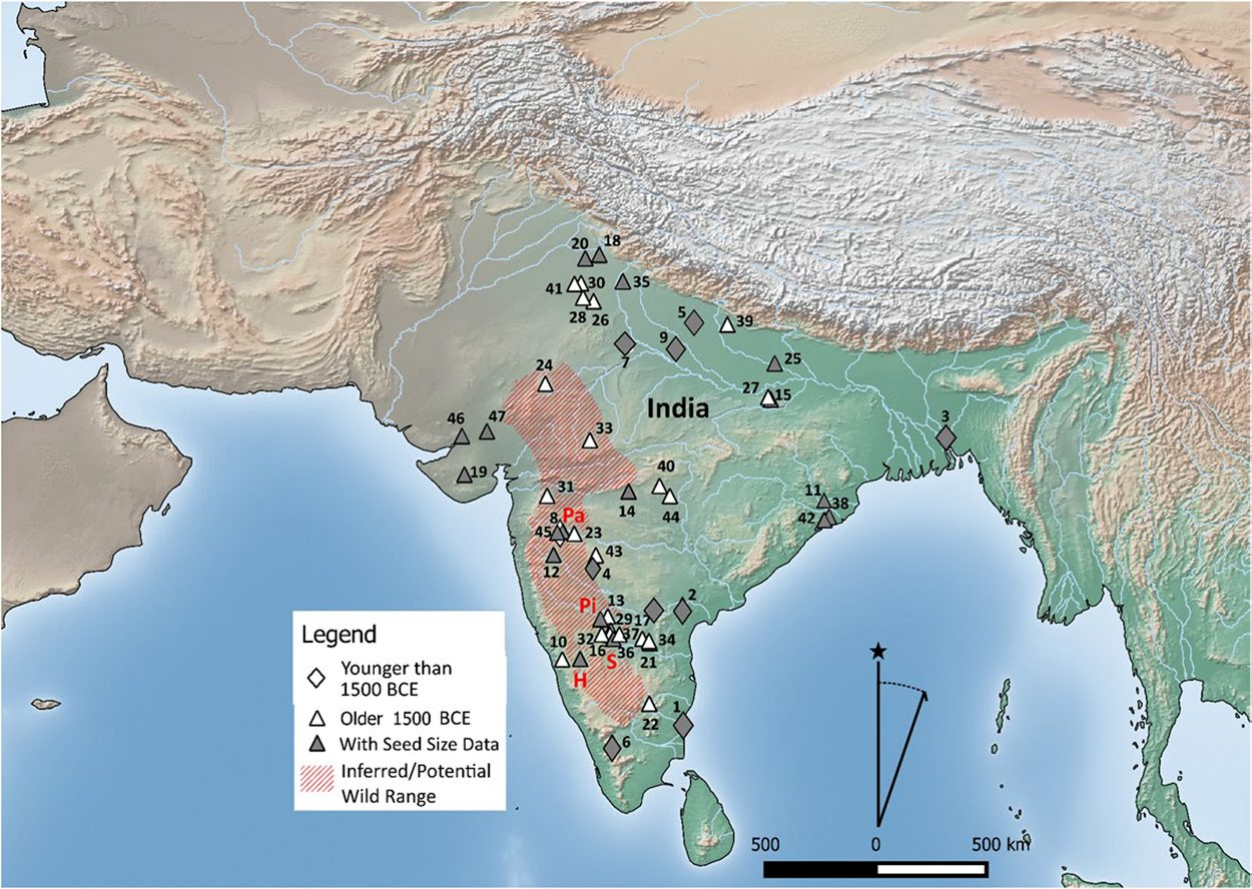
Map showing the distribution of early archaeological horsegram, indicating those sites included in the present study (Pa = Paithan, Pi = Piklihal, H = Hallur, S = Sanganakallu). Additional sites with horsegram recorded (1) Arikamedu (2) Veerapuram (3) Vikrampura Bangladesh (4) Ter (5) Saunphari (6) Perur (7) Noh (8) Nevasa (9) Ahirua Rajarampur (10) Brahmagiri (11) Ostapur (12) Inamgaon (13) Watgal (14) Tuljapur Garhi (15) Tokwa (16) Tekkalakota (17) Singanapalle (18) Sanghol (19) Rojdi (20) Rohira (21) Peddamudiyam (22) Paiyampalli (23) Paithan (24) Ojiyana (25) Narhan (26) Mithathal (27) Malhar (28) Ludwala (29) Kurugodu (30) Kunal (31) Kaothe (32) Kadebakele (33) Kayatha (34) Iinjedu (35) Hulas (36) Hiregudda (37) Hattibelagallu (38) Harirajpur (39) Bhagimohari (41) Banawali (42) Golbai Sassan (43) Apegaon (44) Adam (45) Daimabad (46) Kanmer (47) Lotehswar. Also shown the predicted original wild range. Map created using QGIS Development Team, QGIS 2.12.3- Lyon 2015. QGIS Geographic Information System. Open Source Geospatial Foundation Project. http://www.qgis.org/.

Horsegram is an annual native to South Asia^14^, where it has been an important crop since the beginnings of agriculture in many parts of South Asia. It is the most widely recovered pulse crop on prehistoric or early historic sites in India^15^. Today it is the fifth most widely cultivated grain legume in India, and arguably the most hardy, as well as a key vegetable protein source for hundreds of millions of rural inhabitants on the subcontinent^16, 17^. Evidence from the wild progenitor (*Macrotyloma uniflorum* var. *stenocarpum*) has been poorly studied and has never been described in the floristic studies of India. Thus, the regional origins of Horsegram still remain largely obscure^18^. Recent archaeobotanical sampling in the Deccan plateau of South India, a large, arid region featuring rich Neolithic period remains^5, 18–21^ has shown that a number of the earliest Southern Neolithic crop domesticates, including horsegram, appear to have been locally domesticated. Thus, based upon this evidence and limited assessment of herbarium specimens from the Botanical Survey of India, Pune an inferred possible South Indian origin has been postulated^15^, suggesting that wild horsegram was native to the semi-arid scrub savannah environments ranging from the Acacia thickets from the Aravalli hills in Rajasthan, the savannahs of the Southern Peninsula^22^, while the lower slopes of the western Himalayas are all also plausible foci of early cultivation or domestication (Fig. 1)^23, 24^. Previously, it had been simply assumed that all archaeological examples were domesticated crops, lending speculation to the question of where and when the domestication process took place^25^. In contrast, here we report findings for morphological change dating to this domestication process.

It may be that wild horsegram populations were more widespread during the mid-Holocene climatic wet phase, and the subsequent reduction of their availability, during the aridification that began in the later Fourth Millennium BC, may be connected to their domestication and the emergence of the Southern Neolithic (which coincide largely with the territory of the modern states of Karnataka and parts of Telangana); as these hunter/gatherer/foragers began to collect, concentrate and tend to patches of horsegram in their seasonal rounds^15, 22, 26–28^. Archaeobotanically, horsegram is widely reported and recovered from Chalcolithic and Neolithic sites, with the earliest known occurrence from the site of Khujhun, in the Vindhyan plateau^29, 30^, the Harappan site of Burthana Tigrana in Haryana^31^ and Southern Neolithic sites of Andhra and Karnataka^29, 32, 33^ (Fig. 1). In South India, horsegram is regarded as one of the co-staple crops in an indigenous package of crop domesticates that also included mungbean (*Vigna radiata*) and the millet, browntop millet (*Brachiaria ramosa*)^26^. Together these crops provided the economic base for the emergence of sedentary villages on the Indian peninsula between 5,000 and 4,000 years ago (3,000–2,000 BC)^19, 34, 35^.

Grain size increase is a trait used to measure rates of domestication which has been documented in several pulses including mungbean, lentil, pea, chickpea (*Cicer arietinum*), and soybean (*Glycine max*)^4^. Horsegram metrics from the Deccan region, as well as those from the western states of Gujarat and Rajasthan, indicate that trends towards size increase were underway before 2,000 BC^36^. The timing of size increase appears to finish somewhat earlier in the northwest and this distinct trend suggests that the domestication process was separate and may have begun earlier in northwestern India, perhaps from wild populations in the western Himalayas that were brought down to the Indo-Gangetic plains for cultivation. The current morphometric data on archaeological horsegram^36^ indicates that seed length and width are smaller in the earliest populations and appear to increase in both length and width around the middle of the Second Millennium BC, suggesting that domestication started by at least 4,000 years ago (2,000 BC) and size increase is evident by around 3,500 years ago (1,500 BC) and finishes by 3,000 years ago (1,000 BC). However, seed size change is sometimes regarded as an inaccurate indicator of domestication as this trait may evolve gradually, after, or take longer than, more essential domestication changes associated with seed shattering and germination^5, 13, 37^. There has been discussion as to whether or not size increase occurs during the initial stages of domestication or later during the process^1, 38^. Recent comparative studies suggest that in general seed size increase in pulses and other seed crops occurs during the same domestication episode that saw the evolution of other domestication traits^4, 39^.

**Figure 2:**
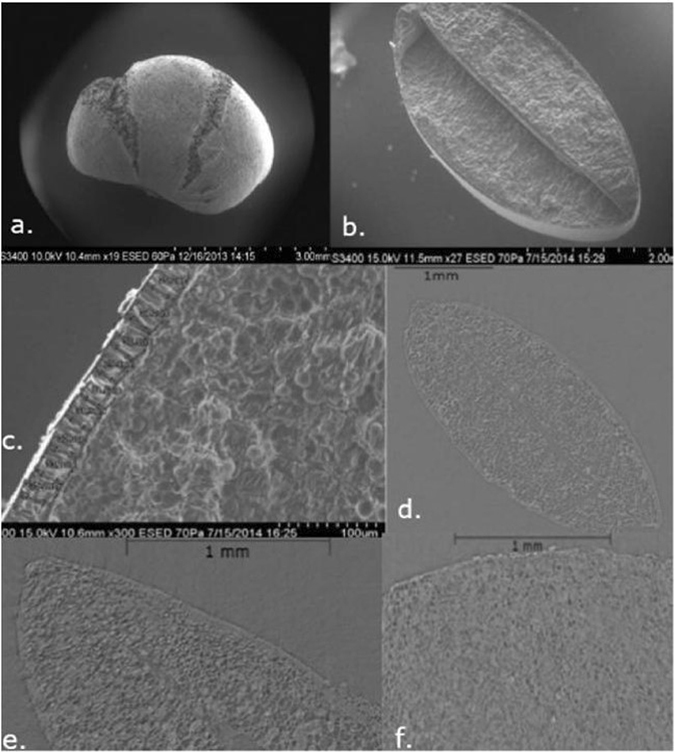
(**a**) SEM of archaeological horsegram (from Sanganakallu) showing a radially fractured seed coat, (**b**,**c**) modern horsegram collected from Dharwad Market, India by author (DQF), a cross section showing cotyledons and seed coat, (**d**–**f**) examples of HRXCT images of archaeological horsegram including (**d**) Domesticated type from the site of Paithan dating to 0–400 AD (Lab code 70019) (**e**) Thicker wild type from the site of Hallur dating to 1,900 BC (Lab code 70018) (**f**) Semi-thin grade from the site of Sanganakallu dating to 1,400–1,250 BC (Lab code 70051).

Loss of germination inhibition, by contrast, is widely regarded as a key feature of domesticated legumes^5, 8–13, 37^. In wild pulses a thick and hard seed coat prevents germination, usually requiring physical damage to allow water to initiate germination. Studies of wild lentil and pea, for example, report germination rates of less than 10%^8, 10, 12^. This means that cultivation of wild-type legumes will offer very poor annual yields if plants are harvested whole and replanted annually. This has led to discussion of the possibility that rare, freely germinating mutants were selected from the wild^8^, rather than evolving under early domestication, and this process, should therefore be, a rapid, consciously-driven selection process^12, 13^. While lentil and pea have been the focus of most studies, and no field trials have been carried out specifically on wild horsegram, one expects parallel evolution in this trait across grain legume crops. However, documenting the evolution of this trait in archaeological seeds has been limited, due to the destruction of the seed coat in preserved archaeological specimens and because, when preserved, observation of the seed coat through conventional methods requires breaking and destroying archaeological specimens. We selected horsegram for this study as we have large assemblages of archaeological horsegram from Indian sites, many of which have wholly or partly preserved seed coats (Fig. 2a), which provided a good test case for methods that can be applied to legume seeds more widely.

## Results

**Figure 3:**
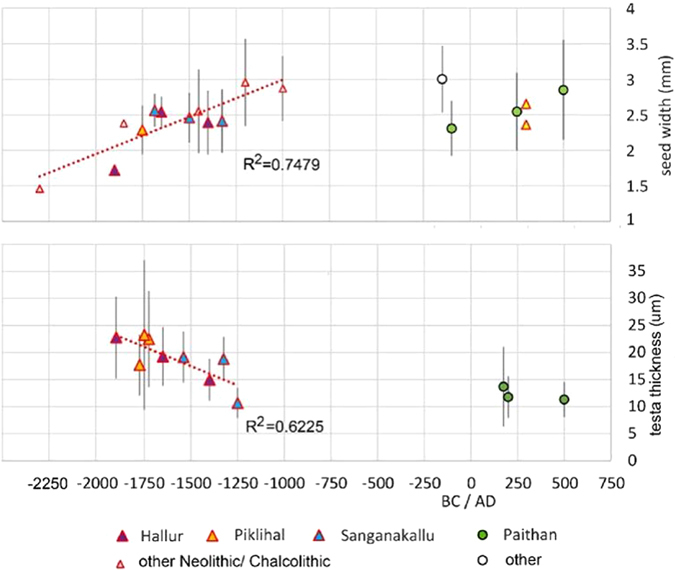
Change in seed size and seed coat thickness over time in Southern Indian archaeological horsegram. Top graph show mean and standard deviation of seed width (data: Table S2). Lower graph shows testa thickness mean and standard deviation (data: Table S3). Trend lines through Neolithic/Chalcolithic samples indicates the trend during the domestication process.

We conducted an experiment using high resolution x-ray computed tomography (HRXCT) to document preserved archaeological seed coats in their entirety and non-destructively. HRXCT and the use of synchrontrons for imaging biological and palaeobiological materials has proved useful in a wide range of studies^40, 41^. Our experiment is the first to try this method for documenting a domestication trait in archaeological seeds. A selection of twelve well-preserved, charred archaeological seeds were selected from four southern Indian sites, Paithan (Pa), Hallur (H), Sanganakallu (S), and Piklihal (Pi) (Fig. 1) representing periods between 2,000 BC to 500 AD (Neolithic to Early Historic periods), which provide a sequence that should capture much of the domestication process in this crop. These were examined at the UK National synchrotron. Three seeds from the early historic period, 0 to 500 AD, should represent fully domesticated forms after the evolutionary domestication process had finished^15^, and therefore represent a control for determining the amount of change that took place earlier. In addition, we measured seed length and width from 450 archaeological seeds from the same sites, combined with 36 published measurements in order to document size change over the same time scale and geography (Tables 1, S2 and S6). These data indicate a significant increase in the average and range of seed width between assemblage before and after ca. 1,700 BC (Fig. 3).

**Table 1:** Seed size measurements on assemblages of archaeological horsegram from various sites and archaeological phases, including seed length (L) and seed width (W) averages and standard deviation.

Site	Source	Period	Length sample size (n)	Median age	L ave	L. stdev	Width n=	Median age	W. ave	W. stdev	Figure 1
Loteshwar	García-Granero 2015	2700–2300 BC	1	−2300	1.69	—	1	−2300	1.46		47
Rojdi	Weber 1991	2000–1700 BC	9 (ave. only)	−1850	3.92	nr	9	−1850	2.38	nr	19
Hallur (98A Layer 8)	This study	ca. 1900 BC	0	—	—	—	1	−1900	1.72	—	H
Piklihal	This study	1900–1600 BC	53	−1750	3.413396	0.409962	104	−1750	2.286731	0.344066	Pi
Sanganakallu	This study	1770–1600 BC	2	−1685	3.305	0.33234	5	−1685	2.564	0.231366	S
Hallur (98A Layer 6)	This study	1800–1500 BC	3	−1650	3.86	0.15	6	−1650	2.54	0.22	H
Sanganakallu	This study	1600–1400 BC	5	−1500	3.534	0.508557	9	−1500	2.458889	0.347687	S
Tuljapur Garhi	Kajale 1996	1700–1200 BC	3	−1450	3.833333	0.650641	3	−1450	2.55	0.589491	14
Hallur (98A Layer 4)	This study	1500–1300 BC	11	−1400	3.62	0.33	17	−1400	2.39	0.45	H
Sanganakallu	This study	1400–1250 BC	191	−1325	3.50487	0.51775	285	−1325	2.413204	0.447362	S
Inamgaon	Vishnu-Mittre and Savithri 1976	1500–900 BC	17	−1200	4.3677	0.74539	17	−1200	2.9559	0.61387	12
Veerapuram	Kajale 1984	1200–800 BC	10	−1000	4.39	0.32515	10	−1000	2.87	0.45521	2
Noh	Vishnu-Mittre 1974	300–1 BC	5	−150	4.35	0.48734	5	−150	3	0.467707	7
Paithan (phase 1)	This study	300 BC–100 AD	20	−100	3.5535	0.5836	19	−100	2.195789	0.366717	Pa
Paithan (phase 2)	This study	0–500 AD	3	250	4.086667	0.553745	3	250	2.93	0.335112	Pa
Piklihal	This study	200–400 AD	—	—	—	—	1	300	2.36	—	Pi
Piklihal	This study	200–400 AD	—	—	—	—	1	300	2.65	—	Pi
Paithan (phase 3)	This study	400–600 AD	0	500	—	—	6	500	2.906667	0.748269	Pa

Seed measurements from this study are provided raw in Table S6. Median age is based upon currently accepted archaeological and radiocarbon information at time of publication^49–53^.

We were able to successfully, non-destructively image archaeological seeds allowing for the first time seed coats to be measured throughout the length and circumference of the seed. An additional result was that we were able to gauge the extent of internal tissue preservation within the seed (Fig. 2d–f)^42^. Our results so far indicate that there is considerable variation within individual archaeological specimens seed coat thickness (Mean values range from 23.2 to 10.6, with corresponding SD 13.8 and 2.8 and SEM 2.2 and 0.47) (Tables S3 and S4), and therefore single spot estimates of testa thickness may be misleading. Some of this variation may be due to differential shrinkage during charring on different parts of the same seed and post-charring damage leading to apparent thinning. This highlights the need for large numbers of testa thickness observations. Despite variation found across individual specimens, there is more variation that is statistically significant between specimens, and this variation indicates temporal trends over time (Figs 3, 5 and 6, Tables 2, 3 and S5).

**Figure 4:**
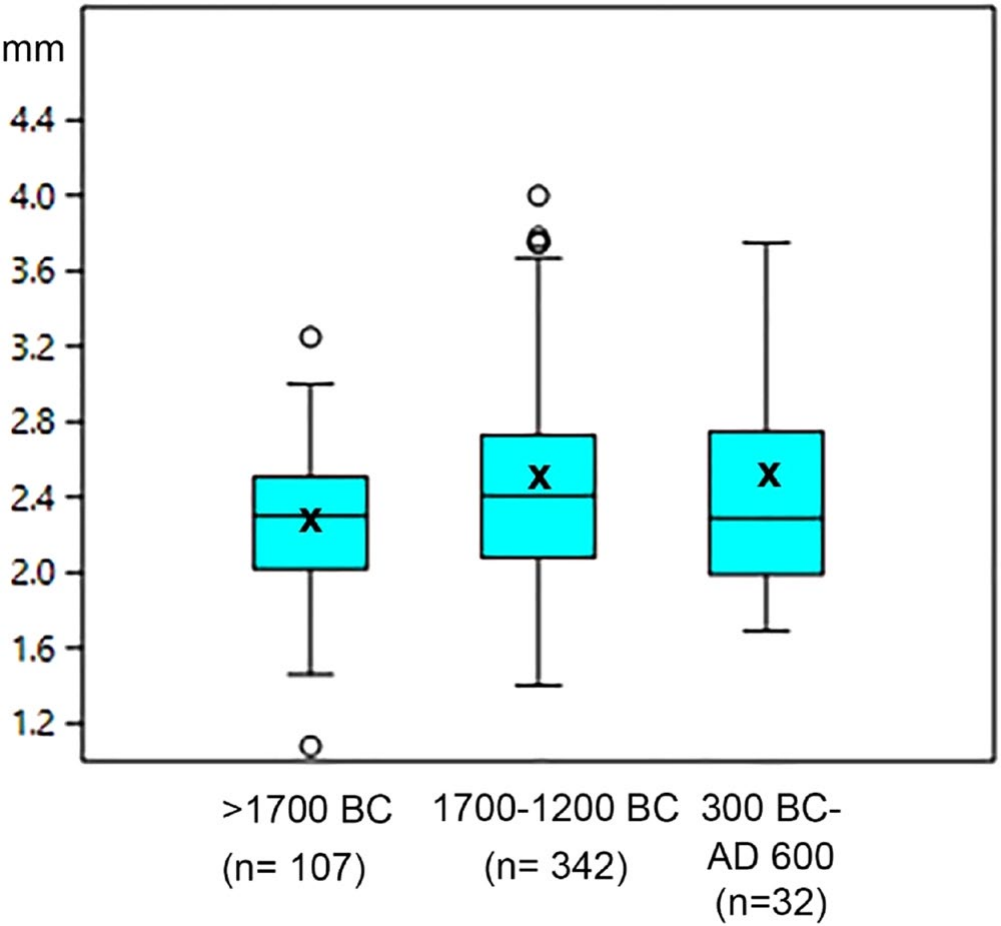
Boxplot of archaeological *Macrotyloma* seed width divided into three periods (raw data in Table S6. X indicates the mean. Means between the early phase (before 1,700 BC) and second phase (1,700–1,200 BC) are significantly different (t-test for equal mean: p = 0.00059786). Kolmogorow-Smirnov test for equal distributions between these two phases is also significantly different (p = 0.0010299).

**Figure 5:**
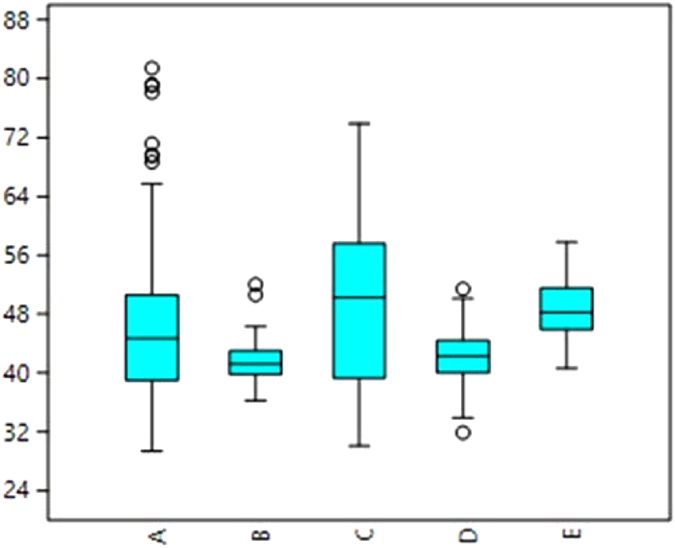
Boxplot of modern, uncharred testa thickness measured in 5 modern domesticated *Macrotyloma uniflorum* var. *uniflorum* populations. Raw data in Table S1.

**Table 2:** Testa thickness measurements on modern horsegram specimens, sectioned and measured under SEM, giving the range of testa thicknesses around the perimeter of a single seed.

	India: Dharwad Market	India: Bellary Market	South Africa: PI 364789 01 SD	Pakistan: PI 365425 01 SD	India: PI 1962290
AVG	45.584	41.627	49.791	42.122	48.713
STDEV	10.395	3.023	11.719	4.084	3.952
MEDIAN	44.7	41.3	48.95	42.3	48.5

The data is presented as mean, standard deviation, and median. Accessions with PI numbers were obtained as germplasm samples from USDA. (For additional individual measurements see Table S1 in data supplement).

**Table 3:** Summary of testa thickness measurements on HRXCT images of archaeological horsegram specimens obtained by synchrotron imaging. Raw measurements in Table S4.

Site Code	Lab Code	Archaeol-ogical Site	Time Period	Median age estimate	Dating Info	AVG Seed coat (micrometers)	STDEV	n	Assigned class
PTN1036	70054	Paithan	AD 400–700	500	Phase 2	11.29	3.21	46	Thin
PTN985	70019	Paithan	AD 0–400	200	Phase 3	11.74	3.87	48	Thin
PTN985	70020	Paithan	AD 0–400	175	Phase 3	13.67	7.37	38	Thin
SAN04 1130	70052	Sanganakallu	1300–1200 BC	−1250	Phase 4	10.66	2.82	41	Thin
SGK98A4-5	70051	Sanganakallu	1400–1250 BC	−1325	Post-Ashmound Pitting/Post-Ashmound village	18.8	4.09	46	Intermediate
HLR98A-4	70027	Hallur	1500–1300 BC	−1400	IIIB	14.96	3.89	41	Thin
SGK98A-6	70049	Sanganakallu	1690–1390 BC	−1540	Post-Ashmound Village/5A	19.15	4.75	40	Intermediate
HLR98A-6	70048	Hallur	1800–1500 BC	−1650	IIIA	19.26	5.42	40	Intermediate
PKL03B 70–100	70055	Piklihal	1900–1600 BC	−1725	Radiocarbon date on Horsegram	22.47	8.9	38	Thick
PKL03B 100–130	70056	Piklihal	1900–1600 BC	−1750	Radiocarbon dates on carbonized seeds (2)	23.23	13.81	39	Thick
PKL03 130–160	70057	Piklihal	1900–1600 BC	−1775	Radiocarbon dates on carbonized seeds (2)	17.68	5.65	43	Intermediate
HLR.98A-8	70018	Hallur	1900 BC	−1900	Radiocarbon date on Horsegram	22.78	7.57	51	Thick
HLR98A-6	70048	Hallur	1800–1500 BC	−1650	IIIA	19.26	5.42	40	Intermediate

The data indicates that we are able to document evidence for morphological evolution in terms of average testa thickness. Seeds can be grouped into two statistically significant testa size grades, thicker (wild type) seed coat that averages above >17 micrometres versus thin testa with averages between 15 and 10 micrometres. Differences between thick/intermediate and thin examples (thin: HLR98A4, SAN04 1130, PTN985, PTN985, PTN1036) are consistently significant to at least p = 0.18 and usually much higher levels (>×10^−5^) (Table S5). However, differences between intermediate and thick testa are only significant in pairwise comparisons between HLR98A-8 and PKL03B 130–160 (p = 0.019), or between PKL03B 130–160 and the other PKL specimens (p = 0.005 and p = 0.04). The whole dataset of thickness measurements is Log^10^ Normal, a common feature of biological and morphological data. Skewness is near 0 or positive with the exception of one slightly negative set (HLR98A-6). Thickness measurements do not fall below 6 micrometers (once charred), which can be regarded as the minimal testa thickness in this taxon. It also appears that the variability within a specimen tends to much greater in the thick and older, and hence more wild, examples, than amongst the intermediate or thinner specimens. This trend deserves further investigation on a larger number of specimens.

**Figure 6:**
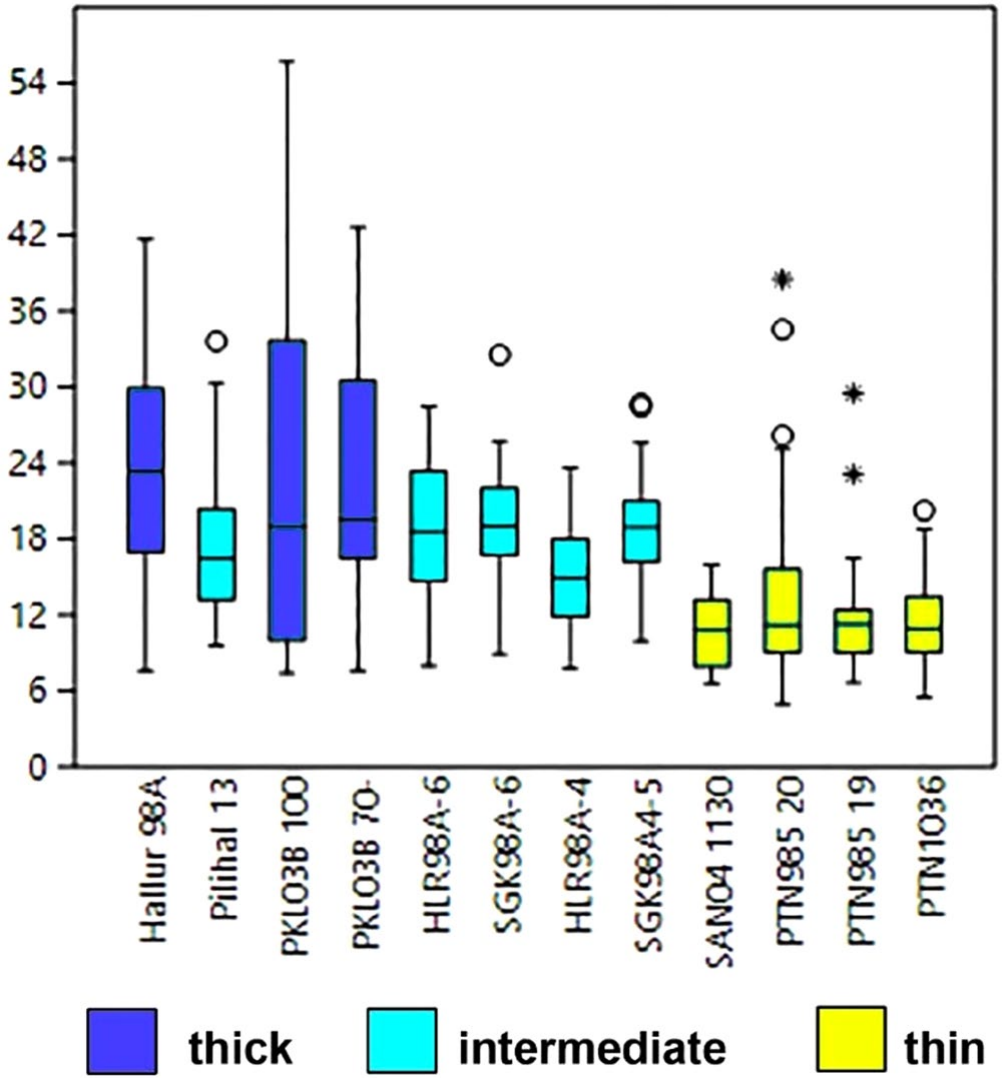
Boxplots of archaeological testa thickness measurements, colour-coded into suggested thick (wild), intermediate and thin (domesticated) testa grades. Boxed areas are the 25th and 75th percentile around the median. Outliers are indicated for measurements at more than 3 times the height of the box; all other measurements fall within the whiskers. Raw data in Table S4.

All of the thinner types are dated to 1,400 BC or more recent, while thicker testa grades are all older than 1,300 BC. Less statistically significant is an intermediate, or semi-thick grade (17–20 micrometers) versus a truly thick, wild type (>22 micrometers) (Fig. 6; Tables 3 and S5). Further samples should help to clarify how robust the thick versus intermediate distinction is. If these are considered, it can be seen that the thickest seed coats predate 1,700 BC, while the intermediate thicknesses have been found between 1,800 BC and 1,300 BC. This would suggest the hypothesis that selection for thinner seed coats, and by inference reduction of germination inhibition, was a stepped process. Of the four earliest specimens dating between 2,000–1,700 BC, three fall in the wild type grade and one in the semi-thin grade. Among the next five specimens dating between 1,700 and 1,250 BC, three are of the semi-thin grade and two of the thin/domesticated type. Finally, three early historic specimens, dating between AD 200 and 500 are all of the thin/domesticated grade. This indicates that the evolution of thinner seed coats took place during the Second Millennium BC and that seed coats were fixed in terms of thickness before the early centuries AD, although further research is needed to identify the morphological status of specimens between 1,250 and 1 BC. However, the directional trend through the Second Millennium BC indicates a rate of morphological change comparable to that estimated for seed coat thinning in North American archaeological *Chenopodium* ^4, 43^. Thus seed coat thinning evolved over a protracted period, as has been documented for other crop domestication traits.

## Discussion

The different grades in testa thickness suggest that this trait is not controlled by a single underlying genetic factor but involves at least two sets of changes. This fits with observations in some other legumes, such as pea and hyacinth bean (*Lablab purpureus*) that show a spectrum of states from high dormancy to easily germinating^10, 44^. Therefore, the directional evolution of the seed coat took place under early cultivation alongside seed size increase (Figs 3 and 4), and this was not more rapid than other domestication traits that have been documented in grain crops^4^. In addition, it implies that cultivation and utilization of this pulse began despite wild dormancy, contrary to predictions proposed for other grain legumes^8, 12, 13^. Horsegram now provides a model for the archaeological documentation of grain legume domestication and affirms the utility of HRXCT for documenting seed coat thickness. This methodology has wider implications and utility for other domesticated crops as it can be applied to other archaeobotanical domesticates to non-destructively assess traits of domestication such as testa thickness and other morphometric properties connected with domestication. It is anticipated that this technique will propel forward research on the biogeographical origins, diversification and improvement of other pulses and domesticated crops and contribute to future issues of agricultural sustainability.

## Methods

Archaeological seed specimens were individually mounted with clear nail varnish on the end of 18 mm cryo-pins (Moloceular Dimensions Limited, MD7-410) and placed in the beamline imaging chamber, Diamond Light Source beamline I-13-2, which is a 250 m long branch-line allowing relatively low energy and large field of view imaging. Samples were each run with 180° scan, with 4,000 projections at 0.15, 0.2 or 0.25 second exposure time each, with 10 darks and 10 flats, with an energy of 15 KeV. We found that 0.15 exposure was sufficient, but 0.2 second had improved resolution. Images were captured with the PCO 4000 detector and 4x objective. We also tried the 10x objective but found that 4x had sufficient magnification and better allowed for the whole seed to be captured.

Tomography was computed with the DAWN software run on the Diamond Light Source servers^45^, which produced between 2000 and 4000 thin section slices across each seed. Seed coat measurements were taken on a selection of 10 slices across the seed, evenly spaced, and 4 measurements were taken at approximately 30°, 70°, 120°, and 180° degrees around the seed circumference. Due to preservation differences between specimens some measurements are absent as part of the seed coat was missing. Measurements were taken with Leica software (LAS-EZ-V3-2-0) and were calibrated for each image based on the width to the cryo-pin.

### Sites

All the synchrotron imaged archaeological horsegram chosen for this study were from South India. Specifically, Piklihal is a well-documented, multi-phase Southern Neolithic period site, consisting of a complex of occupation areas located in the Deccan plateau, with a well-established chronology, based upon radiocarbon dates dating from the Lower Neolithic to the Medieval period^36^. Sanganakallu is a Neolithic village settlement^19, 34^. Hallur is an early Chalcolithic site^19, 26^. Paithan is a much later, well-sampled Early Historic site^46^.

### Dating

The date of specimens were inferred from their archaeological context. The Neolithic sites included in this study were all from South India and included Piklihal, Sanganakallu and Hallur which are all well dated by direct AMS dates on crop remains, coherent stratigraphic relationships and Bayesian statistical models of published absolute chronology^19^. Paithan is an Early Historic site in which the approximate age has been inferred from artefact types including coins.

### Significance testing

Statistical significance of results of testa thickness measurements was performed in the software Past 3.11, 28 including both a one-way ANOVA that indicates the probability of equal means across all the samples is 1.7 × 10–35. Pairwise comparisons across the dataset were explored with the Tukey-Kramer test^47, 48^.

### Future Research

Another round of beam time on beamline I-13 was awarded for May 2017 with Diamond Light Source. We will be targeting archaeological horsegram samples dating between 1,200 BC to 1 BC to fill in this lacuna and increase the sample size of archaeological horsegram testa measurements.

## Electronic supplementary material

Combined list of Supplementary Tables and Dataset in PDF

Supplementary Dataset Tables S1-S6
