# Supplementary material for: Seed coat thinning during horsegram (Macrotyloma uniflorum) domestication documented through synchrotron tomography of archaeological seeds
Source: Sci Rep. 2017 Jul 14;7:5369. doi: 10.1038/s41598-017-05244-w (PMC5511171; doi:10.1038/s41598-017-05244-w)
Supplement: Supplementary file 1 — Combined list of Supplementary Tables and Dataset in PDF [file 41598_2017_5244_MOESM1_ESM.pdf]

Seed coat thinning during horsegam (*Macortyloma uniflorum*) domestication documented through synchrotron tomography of archaeological seeds

Charlene Murphy and Dorian Q Fuller

Supplementary Information [separate files]

Table S1. Testa thickness measurements on modern horsegram specimens, sectioned and measured under SEM, giving the range of testa thicknesses around the perimeter of a single seed, broken need the mid-point.

Table S2. Seed size measurements on assemblages of archaeological horsegram from various sites and archaeological phases.

Table S3. Summary of testa thickness measurements on HRXCT images of archaeological horsegram specimens obtained by synchrotron imaging. Raw measurements in Table S4.

Table S4. Raw seed coat measurements from HRXCT images for each specimen, listed from thinnest to thickest parts of the seed coat. Summary statistics for measurements on each specimen given at the top of the column. First 8 rows (**in bold**) provide summary statistics, followed by raw measurements.

Table S5. Significance test of pairwise differences, using a Tukey-Kramer test. Significance level *p(s)* above the diagonal, and those difference considered significant are shaded. Below the diagonal are Tukey's studentized *q* values in italics.

Table S6. Raw measurements of seed dimensions on archaeological specimens from Piklhal (Pi), Sanganakallu (S), Hallur (H), and Paithan (Pa). **NB:** Due to the variable preservation of the seed coat in these archaeological specimens some measurements around the seed coat are missing.

India: Dharwad Market	India: Bellary Market	South Africa: PI 364789 01 SD	Pakistan: PI 365425 01 SD	India: PI 1962290
**ave: 45.584**	**ave: 41.627**	**ave: 49.791**	**ave: 42.122**	**ave: 48.713**
**stdev: 10.395**	**stdev: 3.023**	**stdev: 11.719**	**stdev: 4.084**	**stdev: 3.952**
**Median: 44.7**	**Median: 41.3**	**Median: 48.95**	**Median: 42.3**	**Median: 48.5**
29.4	36.2	30.1	31.9	40.6
29.8	36.9	37.9	33.9	43.1
29.9	36.9	37.9	34.6	43.2
30.5	38	38.2	36.6	43.4
30.6	38.2	38.5	36.8	43.4
30.7	38.5	39	37.5	44
30.8	39	39	38.4	44.2
30.9	39.3	39.1	38.8	44.4
31.5	39.4	39.3	39.2	44.7
31.6	39.5	39.3	39.7	45.9
31.9	39.7	39.5	39.8	46.5
32	39.8	39.6	40	46.7
32.8	39.9	41.2	40.2	46.8
32.8	40	41.6	40.4	46.8
33	40.1	44.1	40.4	47.2
33.1	40.1	44.2	40.7	47.5
33.1	40.2	47.7	40.9	47.7
33.4	40.3	50.2	41.2	47.9
34.2	40.7	50.3	41.6	48.1
34.2	40.8	53.2	41.6	48.2
35.2	40.9	54.7	41.7	48.8
35.2	41	54.8	41.9	49.6
35.3	41.2	55.1	42.3	49.7
35.3	41.2	55.9	42.4	49.7
35.3	41.4	56.4	42.5	49.7
35.3	41.4	57.6	42.6	49.7
35.4	41.5	58.5	42.6	49.9
35.5	41.6	58.5	42.7	50.6
36.3	42	61.7	42.8	51.1
36.9	42.3	65	43	51.4
37.3	42.3	67.3	43.1	51.5
37.4	42.4	71.1	44.1	52.1
37.7	42.4	72.5	44.1	52.2
38	42.5	73.9	44.4	52.6
38	42.5		44.4	52.9
38.6	42.7		45.8	53.7
38.8	43		46.1	53.9
38.8	43.3		46.6	55.3
38.8	43.5		46.7	56
39	43.6		46.7	57.8
39	43.7		47	
39	43.8		47.1	
39.1	44.3		49.2	
39.2	45.4		50.1	

39.3	45.8	51.4
39.3	46.3	
39.7	50.6	
39.8	52	
39.8		
39.9		
39.9		
40		
40.1		
40.3		
40.4		
40.4		
40.6		
40.6		
40.8		
40.8		
40.9		
40.9		
41.2		
41.2		
41.5		
41.8		
41.9		
42		
42.3		
42.3		
42.8		
42.9		
43		
43.2		
43.2		
43.7		
44		
44.2		
44.4		
44.5		
44.7		
44.8		
44.8		
45		
45.2		
45.2		
45.3		
45.6		
45.6		
45.6		
45.9		
45.9		
45.9		
46.3		

46.4
46.5
46.7
46.7
46.8
47.1
47.4
47.4
47.5
47.6
47.6
47.7
48.2
48.5
48.6
48.9
49.1
49.2
49.2
49.2
49.4
49.5
49.8
49.8
49.9
50
50.3
50.6
50.7
51
51
51
51.2
51.4
51.4
51.5
51.6
51.9
52.5
52.6
52.6
53.9
55.2
55.3
55.3
55.3
55.5
55.6
55.7
56.7

57.4
58.2
58.4
60.9
62.9
63.5
65
65.7
65.7
68.7
69.5
69.7
71.2
78.1
79
79.2
81.4

Site	Source	Period	Length sample size (n)	median age	L ave	L. stdev
**Loteshwar**	**García-Granero 2015**	**2700-2300 BC**	1	-2300	1.69	-
**Rojdi**	**Weber 1991**	**2000-1700 BC**	9 (ave. only)	-1850	3.92	nr
**Hallur (98A Layer 8)**	**This study**	**ca. 1900 BC**	0	-	-	-
**Piklihal**	**This study**	**1900-1600 BC**	53	-1750	3.413396	0.409962
**Sanganakallu**	**This study**	**1770-1600 BC**	2	-1685	3.305	0.33234
**Hallur (98A Layer 6)**	**This study**	**1800-1500BC**	3	-1650	3.86	0.15
**Sanganakallu**	**This study**	**1600-1400 BC**	5	-1500	3.534	0.508557
**Tuljapur Garhi**	**Kajale 1996**	**1700-1200 BC**	3	-1450	3.833333	0.650641
**Hallur (98A Layer 4)**	**This study**	**1500-1300BC**	11	-1400	3.62	0.33
**Sanganakallu**	**This study**	**1400-1250 BC**	191	-1325	3.50487	0.51775
**Inamgaon**	**Vishnu-Mittre and Savithri 1976**	**1500-900 BC**	17	-1200	4.3677	0.74539
**Veerapuram**	**Kajale 1984**	**1200-800 BC**	*10*	-1000	4.39	*0.32515*
**Noh**	**Vishnu-Mittre 1974**	**300-1 BC**	5	-150	4.35	0.48734
**Paithan (phase 1)**	**This study**	**300 BC-100 AD**	20	-100	3.5535	0.5836
**Paithan (phase 2)**	**This study**	**0-500 AD**	3	250	4.086667	0.553745
**Piklihal**	**This study**	**200-400 AD**	-	-	-	-
**Piklihal**	**This study**	**200-400 AD**	-	-	-	-
**Paithan (phase 3)**	**This study**	**400-600 AD**	0	500	-	-

Width n=	median age	W. ave	W. stdev	Map # from Figure 1
1	-2300	1.46	-	47
9	-1850	2.38	nr	19
1	-1900	1.72	-	H
104	-1750	2.286731	0.344066	Pi
5	-1685	2.564	0.231366	S
6	-1650	2.54	0.22	H
9	-1500	2.458889	0.347687	S
3	-1450	2.55	0.589491	14
17	-1400	2.39	0.45	H
285	-1325	2.413204	0.447362	S
17	-1200	2.9559	0.61387	12
10	-1000	2.87	0.45521	2
5	-150	3	0.467707	7
19	-100	2.195789	0.366717	Pa
3	250	2.93	0.335112	Pa
1	300	2.36	-	Pi
1	300	2.65	-	Pi
6	500	2.906667	0.748269	Pa

Site Code	Lab Code	Archaeological Site	Time Period	Median age estimate	Dating Information	AVG Seed coat (μm)	STDEV
PTN1036	70054	Paithan	400-700AD	500	Phase 2	11.29	3.21
PTN985	70019	Paithan	0-400AD	200	Phase 3	11.74	3.87
PTN985	70020	Paithan	0-400AD	175	Phase 3	13.67	7.37
SAN04 1130	70052	Sanganakallu	1300-1200BC	-1250	Phase 4	10.66	2.82
SGK98A4-5	70051	Sanganakallu	1400-1250BC	-1325	Post-Ashmound Pitting/Post-Ashmound Village	18.8	4.09
HLR98A-4	70027	Hallur	1500-1300BC	-1400	IIIB	14.96	3.89
SGK98A-6	70049	Sanganakallu	1690-1390BC	-1540	Post-Ashmound Village/5A	19.15	4.75
HLR98A-6	70048	Hallur	1800-1500BC	-1650	IIIA	19.26	5.42
PKL03B 70-100	70055	Piklihal	1900-1600 BC	-1725	Radiocarbon dates on carbonised seeds x2	22.47	8.9
PKL03B 100-130	70056	Piklihal	1900-1600 BC	-1750	Radiocarbon dates on carbonised seeds x2	23.23	13.81
PKL03 130-160	70057	Piklihal	1900-1600 BC	-1775	Radiocarbon date on Horsegram	17.68	5.65
HLR.98A-8	70018	Hallur	1900BC	-1900	IIIA	22.78	7.57

n	Assigned class
46	Thin
48	Thin
38	Thin
41	Thin
46	Intermediate
41	Thin
40	Intermediate
40	Intermediate
38	Thick
39	Thick
43	Intermediate
51	Thick

Hallur 98A-8	Pilihal 130-160	PKL03B 100-130	PKL03B 70-100	HLR98A-6	SGK98A-6
**Mean: 22.7804**	**17.68004**	**23.23749**	**22.47205**	**19.26017**	**19.15465**
**Std. error: 1.060344**	**0.918012**	**2.211561**	**1.357468**	**0.857011**	**0.770874**
**Median: 23.35675**	**16.38445**	**18.97931**	**19.55115**	**18.70769**	**19.04103**
**25 prcntl: 16.99085**	**13.22968**	**9.997241**	**16.49274**	**14.81026**	**16.67751**
**75 prcntl: 29.9039**	**20.54841**	**33.68828**	**30.50478**	**23.34359**	**22.2459**
**Skewness: 0.071191**	**1.010624**	**0.570653**	**0.52799**	**-0.10202**	**0.164402**
**Excess Kurtosis: -0.57288**	**0.694659**	**-0.78966**	**-0.56196**	**-0.7104**	**0.916472**
**Coeff. Var: 21.4228**	**16.8886**	**19.23636**	**20.75457**	**18.43783**	**18.53818**
7.600573441	9.6	7.404137931	7.600707965	7.964444444	8.922715007
9.673457107	11.19434629	7.404137931	9.61699115	8.752136752	9.896907216
11.21396081	11.30742049	7.80137931	10.74973451	12.03418803	10.20687746
12.04084883	12.01978799	7.955862069	11.32743363	12.08888889	13.37300214
12.81110069	12.01978799	8.033103448	11.32743363	12.47179487	14.76786826
13.20755385	12.17809187	8.827586207	12.91327434	12.47179487	14.76786826
13.78524274	12.23462898	8.893793103	13.68353982	13.83931624	14.88964229
13.83055167	12.49469965	9.103448276	15.40530973	14.22222222	16.11845292
14.19302313	13.22968198	9.103448276	15.40530973	14.22222222	16.46163426
14.89531159	13.22968198	9.997241379	15.47345133	14.6817094	16.74946378
15.20114688	13.61413428	10.1737931	16.49274336	15.19589744	16.86016744
15.40503708	13.61413428	11.72965517	16.84389381	15.31623932	16.89337854
16.99084972	13.76113074	11.88413793	16.84389381	16.41025641	17.18120805
17.33066671	14.48480565	11.88413793	16.99115044	17.63555556	17.32512281
17.9083556	14.74487633	15.68	17.02513274	17.67931624	17.50224867
17.9083556	15.20848057	16.40827586	17.62548673	18.04034188	18.32145575
18.44073556	16.19222615	17.78758621	17.69345133	18.10598291	18.42108905
18.84851596	16.19222615	18.19586207	18.57699115	18.20444444	18.81962222
19.29027805	16.30530035	18.97931034	18.84884956	18.3357265	19.04102954
19.29027805	16.46360424	18.97931034	19.22265487	18.5982906	19.04102954
19.81133077	16.81413428	20.96551724	19.48318584	18.81709402	19.32885906
21.75961487	16.96113074	23.38206897	19.55115044	19.10153846	19.32885906
21.87288721	17.66219081	24.37517241	20.42336283	20.34871795	19.95986992
22.56384843	18.40848057	25.01517241	22.33769912	21.12547009	20.41375493
23.10755562	19.51660777	29.13103448	22.79079646	22.01162393	20.59088079
23.35675475	19.74275618	30.0137931	23.35716814	22.12102564	20.88978067
23.78718961	20.00282686	30.13517241	23.78761062	22.15384615	21.00048433
24.00240704	20.35335689	31.22758621	23.90088496	22.15384615	21.03369543
24.0250615	20.38727915	33.55586207	23.90088496	22.55863248	22.0521691
24.3988602	21.03180212	33.68827586	25.67929204	23.20410256	22.82709472
24.5008053	21.33710247	35.92827586	27.97876106	23.39008547	22.82709472
25.55423798	22.72791519	37.52827586	28.40920354	24.01367521	23.76807583
26.4151077	23.2819788	38.76413793	30.50477876	24.28717949	24.57621255
26.4490894	25.50954064	40.94896552	31.25238938	24.46222222	24.87511243

27.27597742	25.58869258	41.64413793	31.97734513	25.61094017	25.24043451
27.97826587	28.47208481	43.53103448	32.07929204	26.6174359	25.29578634
28.65789986	30.33780919	45.72689655	32.39646018	26.91282051	25.74967135
28.83913559	33.61696113	48.77241379	33.4499115	28.40068376	32.55794645
29.90389551		55.70206897	35.9759292	28.40068376	
29.90389551			36.40637168	28.44444444	
29.99451337			37.50513274		
29.99451337			40.84672566		
30.09645847			42.63646018		
30.39096653					
30.50423886					
30.93467372					
31.97677917					
32.20332383					
34.446116					
35.54485761					
41.68421798					

**HLR98A-4**	**SGK98A4-5**	**SAN04 1130**	**PTN985 20**	**PTN985 19**	**PTN1036**
**Mean: 14.96903**	**18.80003**	**10.66069**	**13.67226**	**11.74178**	**11.29427**
**Std. error: 0.608278**	**0.603629**	**0.440877**	**1.195627**	**0.558829**	**0.474337**
**Median: 14.92967**	**18.95513**	**10.81304**	**11.23039**	**11.36438**	**10.69793**
**25 prcntil: 12.07547**	**16.13913**	**7.953345**	**8.909992**	**9.120548**	**9.103448**
**75 prcntil: 17.54237**	**21.03096**	**13.00858**	**15.92623**	**12.3589**	**13.69103**
**Skewness: 0.216068**	**0.213821**	**0.108383**	**1.849001**	**2.594479**	**0.602027**
**Excess Kurtosis: -0.39649**	**0.298426**	**-1.16438**	**3.607872**	**9.694457**	**0.302465**
**Coeff. Var.: 14.45906**	**18.3497**	**10.28602**	**12.24984**	**11.27709**	**10.85648**
7.761234991	9.950608696	6.586620926	4.921552436	6.663013699	5.517241379
8.365008576	10.96347826	6.674442539	6.958436554	7.901369863	6.433103448
8.573584906	12.44382609	6.674442539	7.387833746	7.978082192	7.062068966
10.12144082	12.68869565	6.674442539	7.707129094	7.978082192	7.062068966
10.12144082	13.54573913	7.025728988	7.707129094	7.978082192	7.404137931
10.35197256	14.08	7.025728988	7.784200385	7.978082192	7.404137931
10.81303602	14.51408696	7.025728988	7.924709762	8.350684932	7.80137931
11.03259005	14.51408696	7.366037736	8.015414258	8.767123288	7.955862069
11.46072041	15.1373913	7.761234991	8.389760528	8.767123288	8.033103448
11.87787307	15.73843478	7.914922813	9.083402147	9.04109589	8.827586207
12.27307033	15.93878261	7.991766724	9.468758602	9.04109589	9.103448276
12.8	16.20591304	8.365008576	9.843104872	9.04109589	9.103448276
12.84391081	16.20591304	8.573584906	9.843104872	9.35890411	9.368275862
13.21715266	16.98504348	8.782161235	9.909165979	9.797260274	9.423448276
13.40377358	17.27443478	9.320068611	9.975227085	9.863013699	9.423448276
13.57941681	17.59721739	9.440823328	10.15139004	10.10410959	9.489655172
14.06243568	17.80869565	10.12144082	10.38260391	10.10410959	10.1737931

14.27101201	17.80869565	10.12144082	10.84503165	10.10410959	10.1737931
14.31492281	17.94226087	10.12144082	11.23038811	10.33424658	10.1737931
14.76500858	18.12034783	10.37392796	11.23038811	10.4	10.1737931
14.9296741	18.25391304	10.81303602	11.70382604	10.95890411	10.1737931
14.9296741	18.42086957	10.86792453	12.11120286	10.95890411	10.40551724
15.24802744	18.95513043	10.97770154	12.16625378	10.95890411	10.47172414
15.52246998	18.95513043	11.19725557	12.88191577	11.28767123	10.92413793
15.72006861	18.95513043	11.30703259	13.30030278	11.44109589	11.03448276
16.20308748	19.21113043	12.0754717	13.61959813	11.64931507	11.25517241
16.3238422	19.43373913	12.27307033	14.47839251	11.64931507	11.52
16.60926244	19.43373913	12.51457976	15.3371869	11.85753425	11.72965517
16.60926244	20.03478261	12.51457976	15.64547206	12.05479452	12.33655172
16.79588336	20.06817391	12.51457976	16.76851087	12.05479452	12.33655172
17.0813036	20.06817391	12.8	18.84943573	12.05479452	12.33655172
18.00343053	20.06817391	13.21715266	18.9375172	12.10958904	12.48
18.16809605	20.45773913	13.21715266	19.59812827	12.10958904	12.8662069
18.26689537	20.52452174	13.21715266	21.02945224	12.25205479	13.32965517
19.41955403	21.00313043	13.97461407	25.10322048	12.25205479	13.47310345
19.79279588	21.11443478	14.06243568	26.17120837	12.25205479	14.34482759
20.24288165	21.49286957	14.31492281	34.54995871	12.39452055	14.34482759
20.24288165	22.53913043	14.76500858	38.53564547	13.19452055	14.34482759
21.12109777	22.91756522	15.24802744		13.3369863	14.38896552
22.84459691	23.16243478	15.29193825		13.95068493	14.51034483
23.64596913	23.48521739	15.98353345		14.03835616	15.00689655
	23.48521739			14.03835616	15.00689655
	24.50921739			14.90410959	15.32689655
	25.62226087			15.49589041	16.40827586
	28.43826087			15.69315068	18.79172414
	28.72765217			16.47123288	20.28137931
				23.12328767	
				29.51232877	

	Hallur 98A-8	Pilihal 130-160	PKL03B 100-130	PKL03B 70-100	HLR98A-6	SGK98A-6	HLR98A-4	SGK98A4-5
Hallur 98A-8		0.01936	1	1	0.3687	0.3217	2.04E-05	0.1902
Pilihal 130-160	*5.031*		0.005989	0.03964	0.9947	0.9971	0.7653	0.9998
PKL03B 100-130	*0.4508*	*5.481*		1	0.1911	0.1603	1.80E-05	0.08343
PKL03B 70-100	*0.3041*	*4.726*	*0.755*		0.5201	0.4666	2.72E-05	0.3022
HLR98A-6	*3.472*	*1.559*	*3.923*	*3.168*		1	0.1105	1
SGK98A-6	*3.576*	*1.454*	*4.027*	*3.272*	*0.1041*		0.134	1
HLR98A-4	*7.705*	*2.674*	*8.155*	*7.4*	*4.232*	*4.128*		0.2403
SGK98A4-5	*3.926*	*1.105*	*4.377*	*3.622*	*0.4538*	*0.3498*	*3.779*	
SAN04 1130	*11.95*	*6.923*	*12.4*	*11.65*	*8.482*	*8.378*	*4.249*	*8.028*
PTN985 70020	*8.984*	*3.953*	*9.434*	*8.679*	*5.511*	*5.407*	*1.279*	*5.058*
PTN985 70019	*10.89*	*5.857*	*11.34*	*10.58*	*7.416*	*7.311*	*3.183*	*6.962*
PTN1036	*11.33*	*6.298*	*11.78*	*11.02*	*7.857*	*7.753*	*3.625*	*7.403*

SAN04 1130	PTN985 70020	PTN985 70019	PTN1036
1.76E-05	1.76E-05	1.76E-05	1.76E-05
7.65E-05	0.1818	0.002063	0.000529
1.76E-05	1.76E-05	1.76E-05	1.76E-05
1.76E-05	1.76E-05	1.76E-05	1.76E-05
1.77E-05	0.005515	2.66E-05	1.91E-05
1.77E-05	0.007324	3.12E-05	1.99E-05
0.107	0.9991	0.5123	0.301
1.83E-05	0.01812	6.88E-05	2.71E-05
	0.6225	0.9998	1
2.97		0.973	0.8868
1.066	*1.904*		1
0.6249	*2.345*	*0.4414*	

Taxon	Time	L (mm)	W (mm)
**Hallur**	1800-1500BC		1.72
**Piklihal**	1900-1600 BC		3
**Piklihal**	1900-1600 BC		2.2
**Piklihal**	1900-1600 BC		
**Piklihal**	1900-1600 BC		
**Piklihal**	1900-1600 BC		
**Piklihal**	1900-1600 BC		2.62
**Piklihal**	1900-1600 BC		1.08
**Piklihal**	1900-1600 BC		2.34
**Piklihal**	1900-1600 BC		2.39
**Piklihal**	1900-1600 BC	3.47	2.56
**Piklihal**	1900-1600 BC	3.46	2.29
**Piklihal**	1900-1600 BC	3.32	2.36
**Piklihal**	1900-1600 BC	2.98	2.04
**Piklihal**	1900-1600 BC		2.3
**Piklihal**	1900-1600 BC	3.75	2.67
**Piklihal**	1900-1600 BC		3.25
**Piklihal**	1900-1600 BC		1.94
**Piklihal**	1900-1600 BC		2.48
**Piklihal**	1900-1600 BC		2.56
**Piklihal**	1900-1600 BC		
**Piklihal**	1900-1600 BC		1.74
**Piklihal**	1900-1600 BC		2.18
**Piklihal**	1900-1600 BC	2.67	2.25
**Piklihal**	1900-1600 BC	3.76	1.55
**Piklihal**	1900-1600 BC		2.97
**Piklihal**	1900-1600 BC	3.62	2.44
**Piklihal**	1900-1600 BC	4.14	2.53
**Piklihal**	1900-1600 BC	3.5	2.38
**Piklihal**	1900-1600 BC	3.34	2.27
**Piklihal**	1900-1600 BC	2.87	1.93
**Piklihal**	1900-1600 BC		2.16
**Piklihal**	1900-1600 BC	3.48	2.34
**Piklihal**	1900-1600 BC		2.28
**Piklihal**	1900-1600 BC		2.62
**Piklihal**	1900-1600 BC		2.14
**Piklihal**	1900-1600 BC		2.32
**Piklihal**	1900-1600 BC		2.6
**Piklihal**	1900-1600 BC	3.92	2.64
**Piklihal**	1900-1600 BC	3.36	2.29
**Piklihal**	1900-1600 BC	3.18	2.59
**Piklihal**	1900-1600 BC	3.55	2.31
**Piklihal**	1900-1600 BC	3.94	2.54
**Piklihal**	1900-1600 BC	4.05	2.92
**Piklihal**	1900-1600 BC		2.02
**Piklihal**	1900-1600 BC	2.78	1.97
**Piklihal**	1900-1600 BC		1.65
**Piklihal**	1900-1600 BC		1.92
**Piklihal**	1900-1600 BC		2.77

**Piklihal**	1900-1600 BC	3.37	2.21
**Piklihal**	1900-1600 BC	3.43	2.38
**Piklihal**	1900-1600 BC		2.39
**Piklihal**	1900-1600 BC		2.53
**Piklihal**	1900-1600 BC		2.81
**Piklihal**	1900-1600 BC		2.78
**Piklihal**	1900-1600 BC		2.13
**Piklihal**	1900-1600 BC		2.13
**Piklihal**	1900-1600 BC		1.8
**Piklihal**	1900-1600 BC	3.59	2.35
**Piklihal**	1900-1600 BC	3.49	2.4
**Piklihal**	1900-1600 BC	3.86	2.5
**Piklihal**	1900-1600 BC	3.92	2.85
**Piklihal**	1900-1600 BC	3.94	2.62
**Piklihal**	1900-1600 BC	3.08	2.27
**Piklihal**	1900-1600 BC	3.5	2.01
**Piklihal**	1900-1600 BC	3.03	1.91
**Piklihal**	1900-1600 BC	3.75	2.07
**Piklihal**	1900-1600 BC	4.29	2.14
**Piklihal**	1900-1600 BC	3.02	2
**Piklihal**	1900-1600 BC	3.31	2.08
**Piklihal**	1900-1600 BC		2.72
**Piklihal**	1900-1600 BC		1.95
**Piklihal**	1900-1600 BC	3.28	2.13
**Piklihal**	1900-1600 BC	3.72	2.6
**Piklihal**	1900-1600 BC	3.64	2.39
**Piklihal**	1900-1600 BC	3.69	2.62
**Piklihal**	1900-1600 BC	3.07	2.41
**Piklihal**	1900-1600 BC	3.76	2.58
**Piklihal**	1900-1600 BC		1.68
**Piklihal**	1900-1600 BC		1.79
**Piklihal**	1900-1600 BC	3.35	2.26
**Piklihal**	1900-1600 BC		2
**Piklihal**	1900-1600 BC	2.91	1.98
**Piklihal**	1900-1600 BC	3.12	2.37
**Piklihal**	1900-1600 BC	2.9	2.01
**Piklihal**	1900-1600 BC		2.38
**Piklihal**	1900-1600 BC		2.7
**Piklihal**	1900-1600 BC		2.51
**Piklihal**	1900-1600 BC		
**Piklihal**	1900-1600 BC	3.25	
**Piklihal**	1900-1600 BC	3.25	
**Piklihal**	1900-1600 BC		2.04
**Piklihal**	1900-1600 BC		2.2
**Piklihal**	1900-1600 BC		2.14
**Piklihal**	1900-1600 BC	2.97	1.83
**Piklihal**	1900-1600 BC		2
**Piklihal**	1900-1600 BC		2.46
**Piklihal**	1900-1600 BC		2.38
**Piklihal**	1900-1600 BC	3.64	2.23

**Piklihal**	1900-1600 BC	2.74	1.89
**Piklihal**	1900-1600 BC		2.44
**Piklihal**	1900-1600 BC	2.6	1.86
**Piklihal**	1900-1600 BC		1.78
**Piklihal**	1900-1600 BC		2.48
**Piklihal**	1900-1600 BC		2.38
**Piklihal**	1900-1600 BC	2.81	2.28
**Piklihal**	1900-1600 BC	3.37	2.34
**Piklihal**	1900-1600 BC	4.12	2.79
**Piklihal**	1900-1600 BC		2.29
**Piklihal**	1900-1600 BC		2.11
**Piklihal**	1900-1600 BC		1.73
**Piklihal**	1900-1600 BC		2.33
**Sanganakallu**	1770-1600		2.43
**Sanganakallu**	1770-1600	3.54	2.56
**Sanganakallu**	1770-1600	3.07	
**Sanganakallu**	1770-1600		2.26
**Sanganakallu**	1770-1600		2.84
**Sanganakallu**	1770-1600		
**Sanganakallu**	1770-1600		2.73
**Hallur**	1800-1500BC	3.84	2.69
**Hallur**	1800-1500BC		2.55
**Hallur**	1800-1500BC		2.87
**Hallur**	1800-1500BC		2.19
**Hallur**	1800-1500BC	4.02	2.48
**Hallur**	1800-1500BC	3.72	2.51
**Sanganakallu**	1600-1400BC	2.98	1.74
**Sanganakallu**	1600-1400BC	4.03	2.72
**Sanganakallu**	1600-1400BC	4.05	2.66
**Sanganakallu**	1600-1400BC		2.19
**Sanganakallu**	1600-1400BC		2.43
**Sanganakallu**	1600-1400BC	3.54	2.56
**Sanganakallu**	1600-1400BC	3.07	
**Sanganakallu**	1600-1400BC		2.26
**Sanganakallu**	1600-1400BC		2.84
**Sanganakallu**	1600-1400BC		
**Sanganakallu**	1600-1400BC		2.73
**Tuljapur Garhi**	1500-1000 BC	3.2	2.05
**Tuljapur Garhi**	1500-1000 BC	4.5	3.2
**Tuljapur Garhi**	1500-1000 BC	3.8	2.4
**Hallur**	1500-1300BC	3.45	2.1
**Hallur**	1500-1300BC	3.42	2.19
**Hallur**	1500-1300BC		3.02
**Hallur**	1500-1300BC		2.07
**Hallur**	1500-1300BC	3.77	2.22
**Hallur**	1500-1300BC		2.75
**Hallur**	1500-1300BC		3.52

**Hallur**	1500-1300BC	3.59	2.29
**Hallur**	1500-1300BC	3.12	2.33
**Hallur**	1500-1300BC	3.3	2.12
**Hallur**	1500-1300BC	3.52	2.23
**Hallur**	1500-1300BC	4.13	2.5
**Hallur**	1500-1300BC	4.11	2.36
**Hallur**	1500-1300BC	3.98	2.39
**Hallur**	1500-1300BC		2.99
**Hallur**	1500-1300BC	3.45	2
**Hallur**	1500-1300BC		1.58
**Sanganakallu**	1400-1250BC	4.64	2.85
**Sanganakallu**	1400-1250BC	3.5	2.14
**Sanganakallu**	1400-1250BC	3.15	2.54
**Sanganakallu**	1400-1250BC	2.71	1.93
**Sanganakallu**	1400-1250BC	2.95	2.11
**Sanganakallu**	1400-1250BC	2.96	1.93
**Sanganakallu**	1400-1250BC		1.9
**Sanganakallu**	1400-1250BC	3.26	2.42
**Sanganakallu**	1400-1250BC		
**Sanganakallu**	1400-1250BC		
**Sanganakallu**	1400-1250BC		
**Sanganakallu**	1400-1250BC	3.27	2.15
**Sanganakallu**	1400-1250BC	3.88	2.41
**Sanganakallu**	1400-1250BC		2.53
**Sanganakallu**	1400-1250BC	2.77	1.92
**Sanganakallu**	1400-1250BC	2.71	2.03
**Sanganakallu**	1400-1250BC	3.88	2.65
**Sanganakallu**	1400-1250BC		2.98
**Sanganakallu**	1400-1250BC		3.1
**Sanganakallu**	1400-1250BC	2.86	1.97
**Sanganakallu**	1400-1250BC	3.59	2.49
**Sanganakallu**	1400-1250BC	2.97	2.07
**Sanganakallu**	1400-1250BC	3.38	2.51
**Sanganakallu**	1400-1250BC	3.04	2.08
**Sanganakallu**	1400-1250BC	2.81	2.03
**Sanganakallu**	1400-1250BC	3.37	2.27
**Sanganakallu**	1400-1250BC	2.88	1.95
**Sanganakallu**	1400-1250BC		2.28
**Sanganakallu**	1400-1250BC		2.67
**Sanganakallu**	1400-1250BC		2.81
**Sanganakallu**	1400-1250BC	4.48	2.62
**Sanganakallu**	1400-1250BC	2.95	1.78
**Sanganakallu**	1400-1250BC	3.46	
**Sanganakallu**	1400-1250BC		2.46
**Sanganakallu**	1400-1250BC		2.44
**Sanganakallu**	1400-1250BC		2.01
**Sanganakallu**	1400-1250BC		2.16
**Sanganakallu**	1400-1250BC	2.53	
**Sanganakallu**	1400-1250BC	2.68	1.67
**Sanganakallu**	1400-1250BC	3.75	2.62

**Sanganakallu**	1400-1250BC		2.24
**Sanganakallu**	1400-1250BC		1.69
**Sanganakallu**	1400-1250BC		2.15
**Sanganakallu**	1400-1250BC	2.8	1.69
**Sanganakallu**	1400-1250BC	3.75	2.5
**Sanganakallu**	1400-1250BC	4.05	2.69
**Sanganakallu**	1400-1250BC	3.34	2.56
**Sanganakallu**	1400-1250BC	3.27	2.05
**Sanganakallu**	1400-1250BC	3.43	2.12
**Sanganakallu**	1400-1250BC		2.27
**Sanganakallu**	1400-1250BC	3.17	1.76
**Sanganakallu**	1400-1250BC	3.5	2.16
**Sanganakallu**	1400-1250BC	3.31	2.17
**Sanganakallu**	1400-1250BC	4.06	2.69
**Sanganakallu**	1400-1250BC	3.59	2.28
**Sanganakallu**	1400-1250BC	3.11	2.16
**Sanganakallu**	1400-1250BC	3.04	2.52
**Sanganakallu**	1400-1250BC	4.15	2.29
**Sanganakallu**	1400-1250BC		2.99
**Sanganakallu**	1400-1250BC		2.28
**Sanganakallu**	1400-1250BC	4.01	2.01
**Sanganakallu**	1400-1250BC	3.4	1.95
**Sanganakallu**	1400-1250BC	3.41	1.69
**Sanganakallu**	1400-1250BC		2.66
**Sanganakallu**	1400-1250BC	3.22	2.4
**Sanganakallu**	1400-1250BC	3.03	2.33
**Sanganakallu**	1400-1250BC	4.5	2.88
**Sanganakallu**	1400-1250BC	3.04	2.01
**Sanganakallu**	1400-1250BC		2.82
**Sanganakallu**	1400-1250BC	3.04	1.84
**Sanganakallu**	1400-1250BC		1.84
**Sanganakallu**	1400-1250BC		2.04
**Sanganakallu**	1400-1250BC		2.54
**Sanganakallu**	1400-1250BC		3.25
**Sanganakallu**	1400-1250BC		3.56
**Sanganakallu**	1400-1250BC	3.39	
**Sanganakallu**	1400-1250BC	3.24	2.48
**Sanganakallu**	1400-1250BC	2.9	1.67
**Sanganakallu**	1400-1250BC	3.52	1.95
**Sanganakallu**	1400-1250BC	3.13	2.05
**Sanganakallu**	1400-1250BC		2.31
**Sanganakallu**	1400-1250BC	2.09	1.92
**Sanganakallu**	1400-1250BC		2.96
**Sanganakallu**	1400-1250BC	3.75	2.32
**Sanganakallu**	1400-1250BC	3.04	1.93
**Sanganakallu**	1400-1250BC	3.03	2.24
**Sanganakallu**	1400-1250BC	3.48	2.7
**Sanganakallu**	1400-1250BC	3.46	2.46
**Sanganakallu**	1400-1250BC	5.04	3.11
**Sanganakallu**	1400-1250BC	3.26	2.33

**Sanganakallu**	1400-1250BC	3.04	1.93
**Sanganakallu**	1400-1250BC	2.59	2.25
**Sanganakallu**	1400-1250BC	3.27	2.28
**Sanganakallu**	1400-1250BC		2.71
**Sanganakallu**	1400-1250BC	3.34	2.08
**Sanganakallu**	1400-1250BC		2.55
**Sanganakallu**	1400-1250BC	4.22	2.5
**Sanganakallu**	1400-1250BC		1.82
**Sanganakallu**	1400-1250BC	4.46	3.14
**Sanganakallu**	1400-1250BC	3.85	2.72
**Sanganakallu**	1400-1250BC	3.86	2.93
**Sanganakallu**	1400-1250BC	4.52	2.78
**Sanganakallu**	1400-1250BC	4.17	2.57
**Sanganakallu**	1400-1250BC	3.81	2.78
**Sanganakallu**	1400-1250BC	3.27	2.39
**Sanganakallu**	1400-1250BC	3.6	2.16
**Sanganakallu**	1400-1250BC	3.72	2.33
**Sanganakallu**	1400-1250BC	3.13	1.86
**Sanganakallu**	1400-1250BC	3.86	2.73
**Sanganakallu**	1400-1250BC	3.25	2.52
**Sanganakallu**	1400-1250BC	4.45	2.81
**Sanganakallu**	1400-1250BC	4.49	3.2
**Sanganakallu**	1400-1250BC	2.5	1.58
**Sanganakallu**	1400-1250BC		2.78
**Sanganakallu**	1400-1250BC	3	
**Sanganakallu**	1400-1250BC	3.5	2.57
**Sanganakallu**	1400-1250BC	3.33	2.29
**Sanganakallu**	1400-1250BC	3.17	1.91
**Sanganakallu**	1400-1250BC	3.41	1.99
**Sanganakallu**	1400-1250BC	4.8	3.38
**Sanganakallu**	1400-1250BC		3.06
**Sanganakallu**	1400-1250BC	3.31	1.98
**Sanganakallu**	1400-1250BC		2.1
**Sanganakallu**	1400-1250BC	4.16	3.21
**Sanganakallu**	1400-1250BC	3.24	2.05
**Sanganakallu**	1400-1250BC	3.03	1.76
**Sanganakallu**	1400-1250BC	2.59	1.44
**Sanganakallu**	1400-1250BC		3.19
**Sanganakallu**	1400-1250BC	2.9	2.03
**Sanganakallu**	1400-1250BC		1.75
**Sanganakallu**	1400-1250BC	3.49	2.04
**Sanganakallu**	1400-1250BC		1.71
**Sanganakallu**	1400-1250BC	2.97	1.78
**Sanganakallu**	1400-1250BC	3.07	1.88
**Sanganakallu**	1400-1250BC	3.41	2.81
**Sanganakallu**	1400-1250BC	3.86	2.72
**Sanganakallu**	1400-1250BC	3.18	2.23
**Sanganakallu**	1400-1250BC	4.45	2.83
**Sanganakallu**	1400-1250BC	4.01	2.43
**Sanganakallu**	1400-1250BC	3.2	1.73

**Sanganakallu**	1400-1250BC	3.57	2.73
**Sanganakallu**	1400-1250BC	4.06	2.56
**Sanganakallu**	1400-1250BC	3.64	2.69
**Sanganakallu**	1400-1250BC		1.56
**Sanganakallu**	1400-1250BC	4.31	3.18
**Sanganakallu**	1400-1250BC	3.42	2.62
**Sanganakallu**	1400-1250BC		2.67
**Sanganakallu**	1400-1250BC	3.75	2.34
**Sanganakallu**	1400-1250BC	3.78	3.09
**Sanganakallu**	1400-1250BC		2.5
**Sanganakallu**	1400-1250BC		2.87
**Sanganakallu**	1400-1250BC		3.05
**Sanganakallu**	1400-1250BC	3.71	2.03
**Sanganakallu**	1400-1250BC	3.56	2.73
**Sanganakallu**	1400-1250BC		2.35
**Sanganakallu**	1400-1250BC		2.88
**Sanganakallu**	1400-1250BC	3.05	2.08
**Sanganakallu**	1400-1250BC	3.07	1.85
**Sanganakallu**	1400-1250BC	3.15	2.21
**Sanganakallu**	1400-1250BC		2.31
**Sanganakallu**	1400-1250BC		2.22
**Sanganakallu**	1400-1250BC	3.04	1.72
**Sanganakallu**	1400-1250BC		2.52
**Sanganakallu**	1400-1250BC		
**Sanganakallu**	1400-1250BC	2.9	1.88
**Sanganakallu**	1400-1250BC		2.49
**Sanganakallu**	1400-1250 BC		3.57
**Sanganakallu**	1400-1250 BC	4.42	2.91
**Sanganakallu**	1400-1250 BC	3.49	2.41
**Sanganakallu**	1400-1250 BC	3.4	2.01
**Sanganakallu**	1400-1250 BC	3.43	2.07
**Sanganakallu**	1400-1250 BC	3.86	2.32
**Sanganakallu**	1400-1250 BC	3.94	2.49
**Sanganakallu**	1400-1250 BC	3.62	2.38
**Sanganakallu**	1400-1250 BC	3.93	2.78
**Sanganakallu**	1400-1250 BC	4.44	3
**Sanganakallu**	1400-1250 BC	3.24	2.08
**Sanganakallu**	1400-1250 BC		3.46
**Sanganakallu**	1400-1250 BC	2.96	
**Sanganakallu**	1400-1250 BC		2.45
**Sanganakallu**	1400-1250 BC		2.5
**Sanganakallu**	1400-1250 BC	4.18	2.13
**Sanganakallu**	1400-1250 BC	3.62	2.66
**Sanganakallu**	1400-1250 BC		2.96
**Sanganakallu**	1400-1250 BC		2.39
**Sanganakallu**	1400-1250 BC		1.81
**Sanganakallu**	1400-1250 BC		2.07
**Sanganakallu**	1400-1250 BC	3.62	2.66
**Sanganakallu**	1400-1250 BC	4.75	3.28
**Sanganakallu**	1400-1250 BC		2.2

**Sanganakallu**	1400-1250 BC		2.72
**Sanganakallu**	1400-1250 BC		2.66
**Sanganakallu**	1400-1250 BC		2.57
**Sanganakallu**	1400-1250 BC		1.92
**Sanganakallu**	1400-1250 BC		2.29
**Sanganakallu**	1400-1250 BC		3.19
**Sanganakallu**	1400-1250 BC		3.78
**Sanganakallu**	1400-1250 BC		2.32
**Sanganakallu**	1400-1250 BC		2.42
**Sanganakallu**	1400-1250 BC	3.71	2.56
**Sanganakallu**	1400-1250 BC	3.6	2.92
**Sanganakallu**	1400-1250 BC		3.67
**Sanganakallu**	1400-1250 BC	4.59	3.42
**Sanganakallu**	1400-1250 BC	2.94	2.01
**Sanganakallu**	1400-1250 BC		2.2
**Sanganakallu**	1400-1250 BC	3.13	2.18
**Sanganakallu**	1400-1250 BC	3.25	1.99
**Sanganakallu**	1400-1250 BC	4.3	
**Sanganakallu**	1400-1250 BC		2.56
**Sanganakallu**	1400-1250 BC		2.78
**Sanganakallu**	1400-1250 BC		2.23
**Sanganakallu**	1400-1250 BC	3.16	1.86
**Sanganakallu**	1400-1250 BC		2.58
**Sanganakallu**	1400-1250 BC	3.1	2.4
**Sanganakallu**	1400-1250 BC	3.31	1.91
**Sanganakallu**	1400-1250 BC	3.01	1.91
**Sanganakallu**	1400-1250 BC	3.34	2.13
**Sanganakallu**	1400-1250 BC	3.29	2.18
**Sanganakallu**	1400-1250 BC		2.26
**Sanganakallu**	1400-1250 BC	3.43	
**Sanganakallu**	1400-1250 BC	2.96	2.23
**Sanganakallu**	1400-1250 BC		1.4
**Sanganakallu**	1400-1250 BC	3.1	1.83
**Sanganakallu**	1400-1250 BC		2.27
**Sanganakallu**	1400-1250 BC	3.48	2
**Sanganakallu**	1400-1250 BC	3.8	2.72
**Sanganakallu**	1400-1250 BC	4.02	2.83
**Sanganakallu**	1400-1250 BC	3.32	2.15
**Sanganakallu**	1400-1250 BC		2.27
**Sanganakallu**	1400-1250 BC	3.31	2.36
**Sanganakallu**	1400-1250 BC	3.15	1.88
**Sanganakallu**	1400-1250 BC	3.3	2.25
**Sanganakallu**	1400-1250 BC	3.49	2.41
**Sanganakallu**	1400-1250 BC	3.4	2.01
**Sanganakallu**	1400-1250 BC	3.43	2.07
**Sanganakallu**	1400-1250 BC	3.86	2.32
**Sanganakallu**	1400-1250 BC	3.94	2.49
**Sanganakallu**	1400-1250 BC	3.62	2.38
**Sanganakallu**	1400-1250 BC	3.98	2.78
**Sanganakallu**	1400-1250 BC	4.44	3

**Sanganakallu**	1400-1250 BC	3.24		2.08
**Sanganakallu**	1400-1250 BC			3.46
**Sanganakallu**	1400-1250 BC	2.96		1.95
**Sanganakallu**	1400-1250 BC			2.45
**Sanganakallu**	1400-1250 BC			2.5
**Sanganakallu**	1400-1250 BC	4.18		2.13
**Sanganakallu**	1400-1250 BC	3.62		2.66
**Sanganakallu**	1400-1250 BC			2.96
**Sanganakallu**	1400-1250 BC			2.39
**Sanganakallu**	1400-1250 BC			1.81
**Sanganakallu**	1400-1250 BC			2.07
**Sanganakallu**	1400-1250 BC			
**Sanganakallu**	1400-1250 BC			
**Sanganakallu**	1400-1250 BC			
**Sanganakallu**	1400-1250 BC	3		1.83
**Sanganakallu**	1400-1250 BC			2.43
**Sanganakallu**	1400-1250 BC	3.73		2.45
**Sanganakallu**	1400-1250 BC			2.74
**Sanganakallu**	1400-1250 BC	3.8		2.97
**Sanganakallu**	1400-1250 BC	3.89		2.99
**Sanganakallu**	1400-1250 BC			3.06
**Sanganakallu**	1400-1250 BC			2.84
**Sanganakallu**	1400-1250 BC	3.75		2.57
**Sanganakallu**	1400-1250 BC			3.29
**Sanganakallu**	1400-1250 BC	3.23		2.07
**Sanganakallu**	1400-1250 BC	2.95		1.65
**Sanganakallu**	1400-1250 BC			2.09
**Sanganakallu**	1400-1250 BC	3.97		3.34
**Sanganakallu**	1400-1250 BC	3.95		2.48
**Sanganakallu**	1400-1250 BC			
**Sanganakallu**	1400-1250 BC			
**Sanganakallu**	1400-1250 BC			
**Sanganakallu**	1400-1250 BC	3.75		2.57
**Sanganakallu**	1400-1250 BC	3.06	1,56	
**Sanganakallu**	1400-1250 BC			2.75
**Sanganakallu**	1400-1250 BC	3.09		1.9
**Sanganakallu**	1400-1250 BC			2.2
**Sanganakallu**	1400-1250 BC	3.82		2.23
**Sanganakallu**	1400-1250 BC			2.42
**Sanganakallu**	1400-1250 BC			2.49
**Sanganakallu**	1400-1250 BC	3.74		2.36
**Sanganakallu**	1400-1250 BC	3.5		2.35
**Sanganakallu**	1400-1250 BC			3.59
**Sanganakallu**	1400-1250 BC	4.14		2.51
**Sanganakallu**	1400-1250 BC	3.9		
**Sanganakallu**	1400-1250 BC	3.01		2.32
**Sanganakallu**	1400-1250 BC	3.47		2.32
**Sanganakallu**	1400-1250 BC	3.62		2.27
**Sanganakallu**	1400-1250 BC			3.02
**Sanganakallu**	1400-1250 BC	3.39		

**Sanganakallu**	1400-1250 BC		2.7
**Sanganakallu**	1400-1250 BC		2.97
**Sanganakallu**	1400-1250 BC		3
**Sanganakallu**	1400-1250 BC		2.49
**Sanganakallu**	1400-1250 BC		2.76
**Sanganakallu**	1400-1250 BC		2.42
**Sanganakallu**	1400-1250 BC		2.62
**Sanganakallu**	1400-1250 BC	3.58	2.37
**Sanganakallu**	1400-1250 BC		2.65
**Sanganakallu**	1400-1250 BC	4.8	2.76
**Sanganakallu**	1400-1250 BC	4.29	3.15
**Sanganakallu**	1400-1250 BC		2.34
**Sanganakallu**	1400-1250 BC		2.66
**Sanganakallu**	1400-1250 BC	3.87	2.65
**Inamgaon**	1500-900 BC		
**Inamgaon**	1500-900 BC	3	2
**Inamgaon**	1500-900 BC	3.25	3
**Inamgaon**	1500-900 BC	3.5	2.25
**Inamgaon**	1500-900 BC	3.75	2.5
**Inamgaon**	1500-900 BC	4	2.5
**Inamgaon**	1500-900 BC	4	3
**Inamgaon**	1500-900 BC	4	3
**Inamgaon**	1500-900 BC	4	3
**Inamgaon**	1500-900 BC	4.25	3.25
**Inamgaon**	1500-900 BC	4.75	3
**Inamgaon**	1500-900 BC	5	3
**Inamgaon**	1500-900 BC	5	1.75
**Inamgaon**	1500-900 BC	5	4
**Inamgaon**	1500-900 BC	5	3
**Inamgaon**	1500-900 BC	5.25	3.5
**Inamgaon**	1500-900 BC	5.25	3.75
**Inamgaon**	1500-900 BC	5.25	3.75
**Veerapuram (average)**	1200-800BC	4.39	2.87
**Veerapuram (min)**	1200-800BC	3.6	2
**Veerapuram (max)**	1200-800BC	4.6	3.4
**Noh**	300-0BC	4	2.5
**Noh**	300-0BC	4	2.75
**Noh**	300-0BC	4	3
**Noh**	300-0BC	4.75	3.75
**Noh**	300-0Bc	5	3
**Paithan (phase 1)**	300AD-0BC	3.82	
**Paithan (phase 1)**	300AD-0BC	4.99	2.16
**Paithan (phase 1)**	300AD-0BC	4.32	

**Paithan (phase 1)**	300AD-0BC	3.77	
**Paithan (phase 1)**	300AD-0BC	3.54	2.3
**Paithan (phase 1)**	300AD-0BC	4.02	2.69
**Paithan (phase 1)**	300AD-0BC	3.52	1.98
**Paithan (phase 1)**	300AD-0BC	3.15	
**Paithan (phase 1)**	300AD-0BC	2.99	1.95
**Paithan (phase 1)**	300AD-0BC	2.83	1.72
**Paithan (phase 1)**	300AD-0BC	3.95	2.25
**Paithan (phase 1)**	300AD-0BC	3.12	2
**Paithan (phase 1)**	300AD-0BC	3.14	2.14
**Paithan (phase 1)**	300AD-0BC	3.44	1.92
**Paithan (phase 1)**	300AD-0BC	2.91	1.81
**Paithan (phase 1)**	300AD-0BC	3.94	2.26
**Paithan (phase 1)**	300AD-0BC	3.09	1.99
**Paithan (phase 1)**	300AD-0BC	2.64	1.69
**Paithan (phase 1)**	300AD-0BC		2.09
**Paithan (phase 1)**	300AD-0BC		
**Paithan (phase 1)**	300AD-0BC		2.75
**Paithan (phase 1)**	300AD-0BC	3.82	2.73
**Paithan (phase 1)**	300AD-0BC		2.29
**Paithan (phase 1)**	300AD-0BC		
**Paithan (phase 1)**	300AD-0BC	4.07	3
**Piklihal (PKL 03B-20-50cm)**	200-400 AD		2.36
**Piklihal (PKL 03B-20-50cm)**	200-400 AD		2.65
**Paithan (phase 2)**	0-500 AD	4.19	2.92

**Paithan (phase 2)**	0-500 AD	3.51	
**Paithan (phase 2)**	0-500 AD	4.56	2.6
**Paithan (phase 2)**	0-500 AD		3.27
**Paithan (phase 3)**	400-600 AD		2.08
**Paithan (phase 3)**	400-600 AD		3.65
**Paithan (phase 3)**	400-600 AD		1.96
**Paithan (phase 3)**	400-600 AD		3.68
**Paithan (phase 3)**	400-600 AD		2.9
**Paithan (phase 3)**	400-600 AD		3.17
